# From physics to fixtures to food: current and potential LED efficacy

**DOI:** 10.1038/s41438-020-0283-7

**Published:** 2020-03-30

**Authors:** Paul Kusuma, P. Morgan Pattison, Bruce Bugbee

**Affiliations:** 1 Crop Physiology Laboratory, Utah State University, Logan, 84341 UT USA; 2 Solid State Lighting Services, Johnson City, 37604 TN USA

**Keywords:** Light responses, Photosynthesis

## Overview

Light-emitting diodes (LEDs) have enabled a historic increase in the conversion of electric energy to photons, but this is approaching a physical limit. The theoretical maximum efficiency occurs when all input energy is converted to energy in photosynthetic photons. Blue LEDs can be 93% efficient, phosphor-converted “whites” 76% efficient, and red LEDs 81% efficient. These improvements open new opportunities for horticultural lighting. Here we review (1) fundamental physics and efficiency of LEDs, (2) the current efficacy of LEDs, (3) the effect of spectral quality on crop yield, and (4) the potential efficacy of horticultural fixtures. Advances in the conversion of photons to yield can be achieved by optimization of spectral effects on plant morphology, which vary among species. Conversely, spectral effects on photosynthesis are remarkably similar across species, but the conventional definition of photosynthetic photons (400–700 nm) may need to be modified. The upper limit of LED fixture efficacy is determined by the LED package efficacy multiplied by four factors inherent to all fixtures: current droop, thermal droop, driver (power supply) inefficiencies, and optical losses. With current LED technology, the calculations indicate efficacy limits of 3.4 µmol J^−1^ for white + red fixtures, and 4.1 µmol J^−1^ for blue + red fixtures. Adding optical protection from water and high humidity reduces these values by ~10%. We describe tradeoffs between peak efficacy and cost.

## Physics

The term efficiency applies to ratios with the same units in the numerator and denominator, which can be expressed as a percentage. LED efficiency describes the optical power output divided by the electrical power input (watt/watt or %). The term efficacy applies to ratios with different units. In horticultural lighting, efficacy refers to micromoles of photon output per second, per watt of input power. Since a watt is a joule per second, this simplifies to µmol per joule. The relationship between photon energy and wavelength is expressed in the Planck–Einstein relation, often just called Planck’s equation. This equation states that energy is inversely proportional to wavelength ($E = \frac{{hc}}{\lambda }$). This equation is used to convert between efficiency and efficacy, and it is used to calculate the maximum possible photosynthetic photon efficacy for a given spectrum.

By converting LED efficiency into efficacy, we get the appropriate units for determining the impact of photons on plants per input electrical power. This follows another physical law called the Stark–Einstein Law, which states that for every photon absorbed, only one molecule can react. This Law can be restated to say that one photon excites one electron. In this paper, photon efficacy is limited to photons between 400 and 700 nm, except in the case of far-red LEDs, where photons from up to 800 nm are included. LED package manufacturers often report efficacy in lumens per watt, because this is a meaningful metric for human lighting, but it is not applicable for horticultural lighting because it is a measure of photons weighted for human vision based on the human eye response to different colors.

[In this paper, LED refers to an LED package, which is the LED chip inside a housing. The housing/packaging enables mechanical and electrical connections to the fixture, provides a thermal path, affects photon distribution, and includes the phosphor layer for white LEDs (see below). LED performance specifications are for LED packages. An LED fixture refers to LED packages integrated into a fixture.]

### Fundamental efficiency of LEDs

The fundamental efficiency of LEDs (LED packages) is the product of the following three sub-efficiencies:

1. Electrical efficiency: the ratio of the emitted photon energy expressed in electron volts to the applied voltage (V_photon_/V_*f*_), affected by internal electrical resistance of the LED.
2. The internal quantum efficiency (photon per electron): the conversion of electrons to photons, affected by non-radiative recombination pathways, including impurities and microphysical defects.
3. Photon extraction efficiency: the ratio of photons that exit the LED semiconductor material to total generated photons, affected by internal reflection and reabsorption. Losses in extracting photons out of an LED package are termed “package losses” within the LED industry. These can vary greatly among LED package types.

White LEDs will also incur phosphor conversion losses which will be discussed later in the paper. For a more comprehensive description of LED efficiency, see ref. ^1^. Incremental improvements have been made to each of the three factors above resulting in a substantial improvement of LED packages over the past 10 years. Now, far-red, red, white, and blue LEDs, respectively, can be 77, 81, 76, and 93% efficient (Table 1).

**Table 1:** Efficiency and efficacy of some common LEDs at 100 mA per mm^2^ (near-optimal efficacy) and a 25 °C junction temperature

LED	Peak wavelength or correlated color temperature	Efficiency (W W^−1^)	Photon efficacy (µmol J^−1^)
Blue	450 nm	0.93	3.5
Green	530 nm	0.42	1.9
Red	660 nm	0.81	4.5
Far-red	730 nm	0.77	4.7
Cool white	6500 K	0.76	2.9
Warm white	2700 K	0.69	2.6

Data derived from company websites (see below). The conversion of efficiency to photon efficacy depends on spectral distribution

### White LEDs

**Fig. 1:**
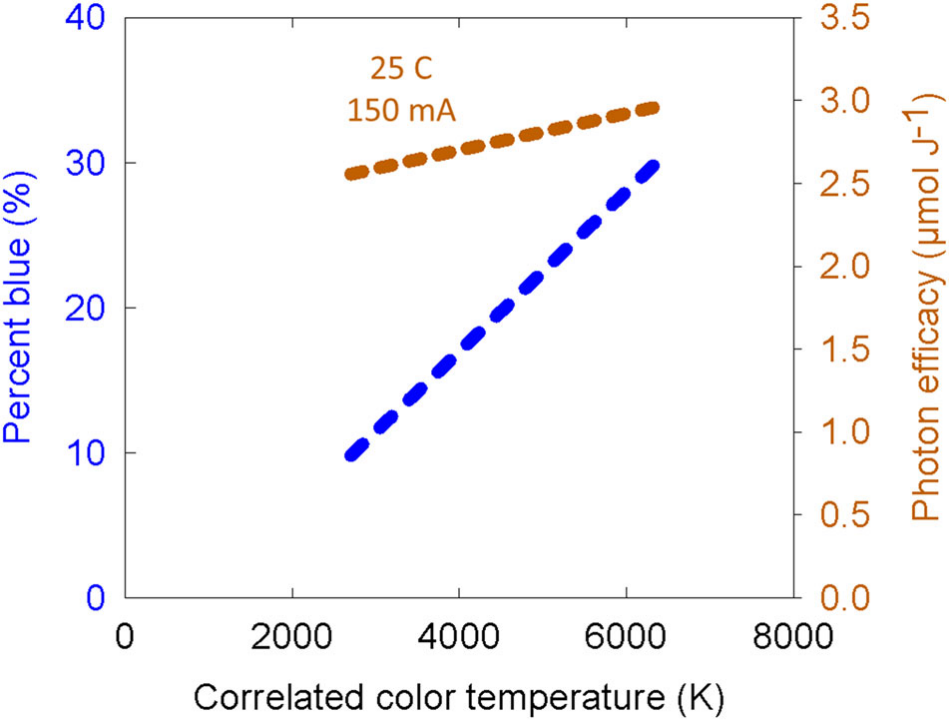
The general relationship between color temperature on percent blue photons (left axis), and the effect of color temperature on photon efficacy (right axis). Exact values vary among manufacturers. Photon efficacy in this graph is presented at a junction temperature of 25°C and 150mA. The efficacy values will shift if these inputs are changed, see below

White LEDs consist of blue LEDs with a luminescent material coating (e.g., a phosphor material, typically Y_3_Al_5_O_12_:Ce) that absorbs blue photons and luminesces at longer wavelengths. Phosphor-converted white LEDs are designed to transmit some blue photons, with the remainder converted to longer wavelengths. Types and amounts of phosphor are varied to create multiple hues and color qualities. Figure 1 shows a general relationship between correlated color temperature (CCT) and percentage of blue photons (400–500 nm). This relationship generally follows Wien’s Displacement Law, which indicates that as the CCT increases the peak wavelength decreases. Therefore, white LEDs with a high CCT have a higher percentage of blue photons. In addition to CCT, electric lights are qualified/quantified by other metrics, including CRI and TM-30 (see ref. ^2^). Both CRI and the TM-30 metric of R_f_ use a scale of 0–100 to describe color fidelity. A high color fidelity facilitates observing subtle color differences. This is important to human observers for visual identification of tiny insects, nutritional disorders, and diseases. Suitable color fidelity is also necessary for machine vision.

Commonly used terms to associate names with color temperatures are warm white (2500–3500 K), neutral white (3500–4500 K), cool white (4500–5500 K), and daylight (5500–7500 K). A lower CCT (2700–4000 K) and higher CRI (greater than 80) are often preferred for indoor lighting to provide incandescent-like light qualities for humans^3^.

Increased density of phosphor coatings and increased use of red phosphor materials decreases efficiency. A 6500 K LED (daylight) with about 30% blue photons can have 95% of the photon output of its non-phosphor-converted blue LED counterpart, but this value decreases to 80–85% for a warm white LED with 10% blue photons. In addition, as the optical output of white LEDs increases, the phosphor efficiency will decrease. This is due to conversion, energy, and optical losses within the phosphor conversion process.

### Current droop

LEDs are designed for performance at specific current ranges. Moderate LED drive current density enables higher efficiency, but at very low drive currents, efficiency decreases. LED loss mechanisms are typically a function of current density. So at a given current, increasing the size of an LED chip can increase the efficacy of an LED by reducing the current density. Unfortunately, chip area is often confidential, and LED manufacturers only report LED specifications at the total LED drive current, not drive current density.

**Fig. 2:**
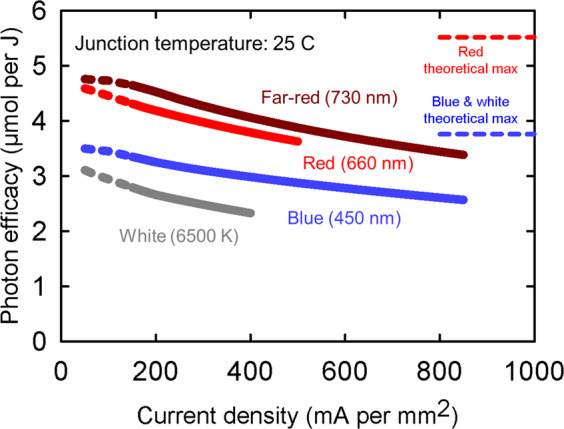
Effects of drive current on photon efficacy at a junction temperature of 25 °C. The dashed lines in this graph represent inadequate test data at low drive currents. However, low drive current (e.g., 65 mA) is used in LED fixtures. Blue photons have a lower theoretical maximum efficacy than red photons, based on Planck’s equation, which states that energy is inversely proportional to wavelength (E = hc/wavelength). Blue photons centered at 450nm can provide 3.76µmolJ^−1^, and red photons centered at 660nm can provide 5.52µmol J^−1^. This is more of a characteristic of the photons than of the LEDs that make them

**Fig. 3:**
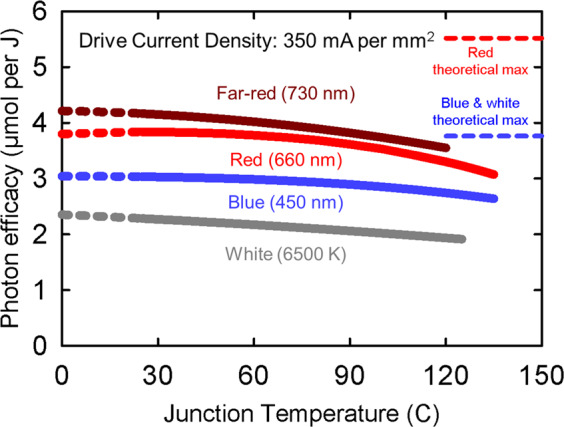
Effects of junction temperature on photon efficacy. Note that higher drive currents increase the junction temperature. The dashed lines in this graph represent temperatures below 25 °C, and therefore temperatures below ambient conditions. Reducing the temperature below ambient would be an energy requiring process

Figures 2 and 3 are calculated using the data from Lumileds (Amsterdam, Netherlands) (Wouter Soer, personal communication)^4^, Osram (Munich, Germany)^5–7^, and Samsung (Seoul, South Korea)^8^. These companies provide LED efficacy data in µmol J^−1^. Additional LED manufacturers include Nichia (Anan, Japan)^9^, Cree (Durham, North Carolina, USA)^10^, Epistar (Hsinchu, Taiwan)^11^, and many others. As technology improves, see the companies’ websites to find the latest LED package efficacy information, and apply the principles described below to determine potential fixture efficacy. Figure 2 shows the decrease in efficacy as a function of drive current density for typical LEDs. This effect is referred to as current “droop”. Current droop is the decrease in radiative efficiency of the LED as current is increased. For a blue LED current, droop is caused by Auger recombination^12,13^. For a white LED, which has a blue LED and a phosphor conversion layer, the droop is caused by Auger in the blue LED and reduction in phosphor conversion efficiency at higher optical flux concentrations^14^. For red and far-red LEDs, the current droop is caused by carrier leakage due to poor confinement of electrons in the active regions^15^. In general, decreasing the drive current increases the efficacy, but eventually Shockley–Read–Hall defect losses will dominate at very low drive current^16^. LED manufacturers continue to both increase LED peak efficiency and reduce current droop. The theoretical maximum lines for red (centered at 660 nm) and blue (centered at 450 nm) LEDs are based on the assumption of 100% power efficiency of the LED or hypothetical photon generating device (1 W electricity input = 1 W photon output) followed by a conversion to number of photons using Planck’s equation. White LEDs would be as efficient as blue LEDs if phosphor conversion was 100% efficient. However, phosphor conversion efficiencies range from 80 to 95%, depending on amount of phosphor, phosphor material, temperature, and photon flux density. A color-mixed “white” fixture using direct-emitting (not phosphor converted) green LEDs (550 nm) as well as red and blue LEDs would have a theoretical maximum of ~4.6 µmol J^−1^, but direct-emitting green LEDs currently have low efficiency (referred to as the green gap^17^) resulting in low efficacy (about 1.9 µmol J^−1^)^18^.

### Thermal droop

Junction temperature refers to the operating temperature at the actual diode. There are two temperature standards for reporting the efficacy of LEDs: 25 and 85 °C. Efficacy decreases ~10%, as the temperature increases from 25 to 85 °C (thermal droop). Thermal droop is typically worse in red compared with blue LEDs.

### Projected efficacy

**Fig. 4:**
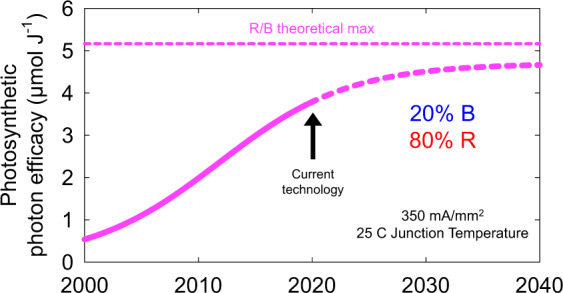
Historical, current, and projected LED package combination efficacy of a 20/80% ratio of blue and red LEDs. This is a weighted average of the two LEDs. The figure constrains LED performance to specific current and temperature operating conditions as discussed above. In practice, the current technology point is not constrained to these conditions so it can be higher than what is shown in the figure

A timeline of the historic and projected increases in LED efficacy is presented in Fig. 4 (see ref. ^19^).

Figure 4 includes an estimate the current efficacy of LED technology. This estimation indicates LED efficacy is approaching a practical maximum. The theoretical maximum assumes 100% efficiency, but is difficult to attain. Therefore efficacy is expected to level off at a practical maximum that is 90% of the theoretical maximum.

## LEDs for horticultural applications

Horticultural LED fixtures typically contain combinations of red (peak ≈ 660 nm), blue (peak ≈ 450 nm), white, and/or far-red (peak ≈ 730 nm) emitting LEDs. Other peak wavelengths are available, but they have lower efficiencies and efficacies and are less common. Fixture manufacturers choose the ratios of these LEDs for specific applications and based on their perception of best practices and market demands.

### History of horticultural LED fixtures

Morrow discussed the significance of LEDs for horticultural lighting and reviewed the early adoption of the technology^20^. The first LED-produced photons used to grow plants were red^21^, which was shortly followed by the development of high output blue LEDs^22^. For a review of the historical significance of this Nobel prize-winning discovery, see ref. ^23^. Before widespread adoption of blue LEDs, early studies demonstrated the value of blue photons for plant growth using blue fluorescent fixtures to supplement red photons from LEDs^24,25^.

The first commercial horticultural LED fixtures were blue + red combinations. These fixtures, which produced a spectrum that appeared magenta, had a higher efficacy than white or white + red fixtures. Many people thought that these blue + red fixtures would enhance photosynthesis compared with full spectrum fixtures due to their close match to the chlorophyll absorption spectrum, which shows peak absorption in the blue and red regions of the photosynthetically active radiation (PAR, 400–700 nm) spectrum. This thinking was advanced by early LED manufacturers, even though green photons have long been known to be effective for photosynthesis^26,27^. Due to widespread use in lighting applications for human vision, white LED packages are now ~20% of the cost of red LEDs. This has contributed to the increase in the fraction of white LEDs to more than 60% in some horticultural fixtures.

### Spectral effects on plant shape and photosynthesis

Photons excite electrons and photobiology is thus driven by the number of photons, not energy or lumens. Biologically active photons must have sufficiently high energy to excite pigment photoreceptors, and there are multiple photoreceptors with weighting functions for wavelengths, which are biophysically or empirically derived. Lumens are an example of a weighting function applied to a photon flux and spectral distribution for human visual function.

The effect of spectral quality on plant shape is synergistic among wavelengths, interacts with intensity, varies among species^28^, and may vary over the plant life cycle. Some principles, however, apply across all species. The impacts of spectrum on plant growth and development are much greater in sole-source lighting than in greenhouse supplemental lighting where electric lighting makes up only a small portion of the plant lighting diet.

In plant biology, spectra are traditionally separated into the following coarse categories.

**Ultra-violet photons** are further separated into three broad categories: UV-C (100–280 nm), UV-B (280–315/320 nm), and UV-A (315/320–400 nm). The wavelength at which UV-C and UV-B are separated (280 nm) is determined by the shortest wavelength of solar radiation that reaches the surface of Earth. The wavelength at which UV-B and UV-A are separated (315 or 320 nm) is generally determined by the effect of sun on human skin sunburn (315 nm) or skin cancer (320 nm). There is no universal agreement on the wavelength transition between UV-B and UV-A, both are equally used. Fortunately, UV-C photons are completely blocked by our atmosphere because they are highly damaging to biological organisms. UV-B photons are also damaging, but can have beneficial effects including increased production of secondary metabolites^29^. UV-A photons are less damaging than UV-B, and can have either stimulatory or inhibitory effects on plant growth, depending on species and interacting environmental factors^30^.

At 25 °C and 350 mA, UV-B and UV-C LEDs are only ~3% efficient^31^, but these photons can have large biological effects in small quantities. At 25 °C and 700 mA, the efficiency of UV-A LEDs increases from 50 to 60% as the wavelength increases from 370 to 395 nm^32^. A violet LED with a peak between 402 and 408 nm is ~65% efficient, and has 15–30% of its photons below 400 nm. Efficiency will increase as current density decreases.

Based on studies by McCree^26,27^, PAR only includes photons with wavelengths between 400 and 700 nm. However, McCree’s studies show significant differences in the photosynthetic efficiency of species at wavelengths below ~425 nm. Some species, like radish, have equal photosynthesis between 375 and 500 nm. Photons between 350 to 400 nm can be photosynthetic, but a high fraction are typically absorbed by non-photosynthetic pigments.

**Blue photons** (400–500 nm) reduce plant height and leaf expansion in nearly all species^28,33–35^. Because of absorption by inactive pigments (e.g., anthocyanin), blue photons are ~20% less photosynthetically efficient than photons from the most common red LED (660 nm)^26,27^. However, the blue-induced decreases in leaf area (reducing photon capture) may have a larger effect on overall plant growth than the blue-induced reduction in photosynthetic rate^28^. A range of 5–30% blue is typically used in horticultural LED fixtures to inhibit excessive stem extension and reduce plant height, which is typically beneficial for controlled environment growth.

**Green photons** (500–600 nm) improve human perception of color. Unfortunately, monochromatic direct emitting (non-phosphor converted) green LEDs have low efficacy. White (phosphor-converted blue) LEDs are thus used to provide the green photons that are important to human vision; and they have the added benefit of providing blue and red photons. Green photons are up to 10% less photosynthetically efficient than photons from the most common red LED (660 nm)^26,27^, but they penetrate deeper into plant canopies than blue or red photons^36^.

The effect of green photons on plant shape is generally much less than the effects of blue or far-red photons. Studies in *Arabidopsis* suggest that green photons can reverse blue photon effects (e.g., inhibition of hypocotyl elongation)^37,38^ or induce shade avoidance (e.g., increased stem elongation, reduced branching)^39,40^. Some studies suggest that green-induced shade avoidance also occurs in food crops and other economically valuable plants^28,35,41^, but several other studies have shown minimal effects^28,33,41–45^.

**Red photons** (600–700 nm) are well absorbed by leaves, are photosynthetically efficient, and are efficiently generated by LEDs so they are widely used in horticultural fixtures. The classical paradigm has been that red and far-red act antagonistically to inhibit or induce shade avoidance symptoms, such as stem elongation, hyponastic leaf orientation, and/or reduced branching^46,47^. However, the high absorbance of red photons by chlorophyll means that the impact of red on shade responses may be overestimated^48^. Replacing green photons with red photons has minimal effects on plant shape^43,45^, but plants grown in the complete absence of red and green photons (sole-source blue LEDs) can rapidly elongate^28,33,49^.

**Far-red photons** (700–800 nm) can have powerful effects on plant shape, and are efficiently generated by LEDs so they are a promising addition to horticultural lighting. Along with several other laboratories, we are working to quantify the effects of far-red photons on plant morphology. In some species (especially lettuce), far-red photons beneficially increase leaf expansion, but they also significantly increase stem elongation in many other species^35,50^, which may not be beneficial.

Despite the classic definition of PAR, recent studies indicate that far-red photons (700–750 nm) are photosynthetically synergistic with shorter wavelength photons^51,52^. These photons are thus being reconsidered for their role in photosynthesis. Far-red photons must be used with caution, particularly in sole-source environments, because they can induce stem elongation associated with shade avoidance.

## Technology of LED fixtures

### Four factors that determine fixture efficacy

The upper limit of fixture efficacy is determined by the choice of LEDs and operating conditions. Different colors of LEDs have different efficacies, but the quality of LEDs within and among manufacturers also varies. In addition to the efficacy of the selected LEDs, fixture efficacy is determined by four additional factors^53^:

1. LED drive current
2. LED junction temperature
3. Driver efficiency
4. Optical losses in the fixture

**1. Drive current**

The effect of drive current on LED efficacy has been discussed above. Due to different types of efficiency reductions at high and low drive currents, efficiency can be maximized at some relatively low drive current (less than 100 mA). Fixture manufacturers seek to co-optimize fixture size, cost, output, and efficacy for specific applications. While low drive currents will increase the efficacy of the fixture, the output of each LED would be relatively low. This increases the cost and complexity of the fixture.

**2. LED junction temperature**

**Fig. 5:**
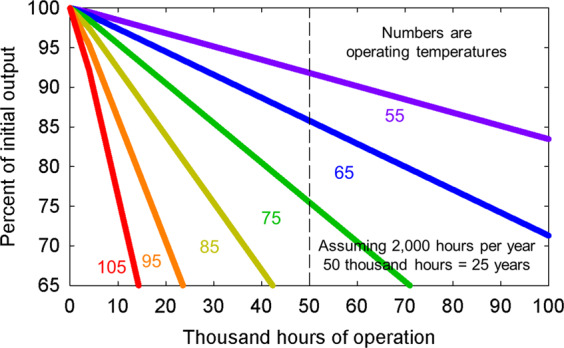
Example long-term depreciation of LEDs based on temperature. LEDs will depreciate slower when operated at lower temperatures. Specific rates of depreciation for LEDs will depend on color, current operation, package type, and LED caliber as well as temperature. Colors on the graph represent temperature and not LED color

The junction temperature of LEDs in fixtures depends on the drive current, ambient temperature, and the heat dissipation (thermal management) of the fixture, but is typically around 85 °C^53^. Better thermal management may increase fixture cost, but it also increases efficacy and longevity of the LEDs (Fig. 5).

[LEDs degrade with time as a function of temperature and current density. L70 is a metric that indicates the time at which a fixture output is 70% of the original output (sometimes referred as Q70 for horticultural products). A typical L70 for LED fixtures is 50,000 h. As the LEDs and fixture age, the efficacy will decline; a problem that is exacerbated by high junction temperatures. Rates of fixture aging can vary greatly among manufacturers. Most manufacturers characterize their fixture lifetime (L70, L90, Q70, or Q90) in terms of LED output depreciation based on a standard LED package test—IES LM-80, which can be interpolated into luminaire lumen maintenance. Projections of luminaire lumen maintenance based on LED depreciation cannot exceed six times of the duration that the LEDs were tested, so for a depreciation lifetime claim of 60,000 h the LEDs must have been tested for 10,000 h. Many fixtures that claim extended lifetimes are exceeding the allowable six times interpolation based on LED testing. Fixture lifetimes based on LED depreciation also do not include optical loss mechanisms in the fixture and accelerated aging of the LEDs due to higher temperatures. Also, typical lifetime claims do not consider catastrophic failure of the LED driver, which often fails before the LEDs have reached the L70.]

**3. LED drivers** (also called power supplies) are necessary to convert AC to DC power and provide regulated voltage and current. The efficiency of LED drivers ranges from 85 to 95%. LED drivers can be less efficient when they provide dimming, color control and/or communication functionality.

**4. Optical losses** occur when LEDs are mounted in fixtures. The sides of the fixture can obstruct low-angle photons. Protective transparent covers (e.g., glass) transmit up to 92% of the photons and thus reduce the output by 8%, but this protection can significantly improve the lifetime of a fixture. Fixtures with unprotected LEDs can have 99% optical efficiency, but may have shorter lifetimes in harsh growing environments (e.g., high humidity).

Optical covers can also diffuse the photons, which reduces efficiency, but can result in more uniform mixing of colors and improved photon penetration into plant canopies^54–56^.

Photons must impact leaves to be absorbed, and this is an important consideration in fixture design. Early LED fixtures had focused photon output over a small area. This facilitated precise photon placement, but caused nonuniform distribution. LED package and fixture design has transitioned to a less-focused photon distribution, but as long as the photons exit the fixture this does not affect our analysis of optical efficacy^57^.

### Potential fixture efficacy

Using the following near-maximum parameters, we now calculate an achievable fixture efficacy using presently-available technology:

1. Drive current minimized to achieve 104% of the reported LED efficacy (100 mA mm^−2^ to 50 mA mm^−2^).
2. Temperature rise minimized by sufficient heat dissipation (e.g., water cooling) to achieve 95% of the reported LED efficacy (at 25 °C).
3. The LED driver is 95% efficient.
4. Unprotected LEDs in the fixture to achieve 99% optical efficiency.

The resulting fixture efficacy would be 1.04 × 0.95 × 0.95 × 0.99 = 93% of the reported efficacy of the LED. The first two factors can be above 100%, if the LEDs are operated at lower drive current and lower temperature than the reported specification (100 mA mm^−2^ and 25 °C here). Reducing drive current is much easier than reducing temperature.

Accordingly, a horticultural fixture with 90% red and 10% blue photons (i.e., a photon flux distribution of B10:R90, typical of magenta-colored fixtures) could potentially achieve an efficacy of 4.1 µmol J^−1^, if it used red and blue LEDs with efficacies of 4.5 and 3.5 µmol J^−1^, respectively (Table 1).

A fixture built using all white LEDs with an efficacy of 2.9 µmol J^−1^ (Table 1) would result in a fixture efficacy of 2.7 µmol J^−1^.

**Fig. 6:**
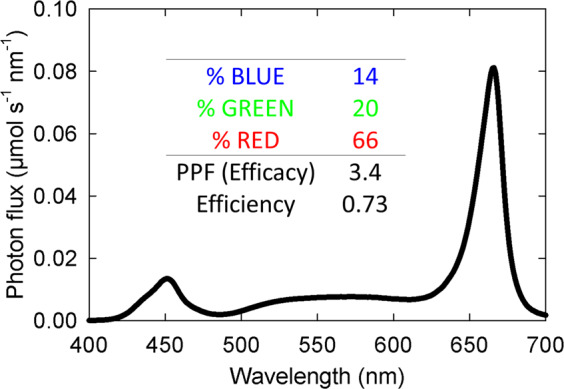
A suggested fixture for high efficacy. The *Y*-axis assumes one watt of input power

A broad spectrum fixture, with approximately equal portions of red and white LEDs could achieve an efficacy of 3.4 under optimal conditions if the best LEDs are used (Fig. 6). The increased leaf expansion caused by far-red photons means that the addition of up to 30% far-red LEDs might be cost effective for lettuce and other leafy greens^35^.

### Typical fixture efficacy

Using more typical parameters:

1. Drive current achieves 90% of the reported LED efficacy.
2. Temperature management achieves 90% of the reported LED efficacy (at 25 °C).
3. The LED driver is 90% efficient.
4. Protected LEDs in the fixture achieve 92% optical efficiency.

The resulting fixture efficacy would be 0.90 × 0.90 × 0.90 × 0.90 = 67% of the reported efficacy of the LEDs.

In 2014, the best LED fixtures had an efficacy of 1.7 µmol J^−1^ (ref. ^57^).

Now, fixture efficacies of 2.5 to 2.8 µmol J^−1^ for white + red fixtures and 3 µmol J^−1^ for blue + red fixtures have been achieved (Table 2).

**Table 2:** Examples of the highest efficacy values from independently-tested LED fixtures and an HPS fixture

Color	Efficacy (µmol J^−1^)	References
Blue/red	2.55	Johnson et al.^64^
Blue/red	2.64	Radetsky^65^
White/red	2.59	Radetsky^65^
Blue/red	3.0	DLC^58^
White/red	2.78	DLC^58^
White/red	2.61	DLC^58^
3000 K	2.13	TÜV SÜD America (2019)
5000 K	2.43	TÜV SÜD America (2019)
1000 W double-ended HPS	1.72	Radetsky^65^

TÜV SÜD America is an accredited testing laboratory

Certified test laboratories conduct comprehensive tests on fixtures to characterize their performance. This is the integrated measure of all the above factors. Fixture manufacturers should always be able to provide test results for their fixtures from certified third-party test laboratories.

The Design Lighting Consortium (DLC) maintains a list of horticultural lighting products that meet their listing requirements^58^. The DLC requires that products have a minimum efficacy of 1.9 µmol J^−1^ and meet photon flux maintenance, driver lifetime, warranty, and safety requirements.

## Additional considerations

In addition to efficacy, several other factors affect fixture choice, including:

1. Initial fixture cost per photon s^−1^ of output capacity. HPS fixtures (1 kW) range from $200 to $350 USD per kW (5–9 µmol s^−1^ per $). LED fixtures range from $1000 to $3000 USD per kW (0.5–1.5 µmol s^−1^ per $). On a photon flux basis, the initial cost is therefore, 3–18 times higher for LED vs. HPS fixtures. The cost of both technologies has decreased, but the cost of LED fixtures are expected to decrease faster than HPS fixtures. While initial cost is higher, LED fixtures reduce energy cost compared with HPS. Depending on usage periods and price of energy, the electric savings can equal the difference in initial cost after of 3–5 years for sole- source applications and 5–8 years for supplemental applications^57^.
2. Spectral quality for plant morphology and photon capture^35,59^.
3. Adequate green photons to create “white” light to facilitate human comfort and visual identification of insects, diseases, and nutritional disorders^60^.
4. Fixture reliability, including environmental protection of the LEDs.
5. Fixture operating temperatures, which affect LED and system longevity.
6. Uniformity and distribution of photon output. Many early LED fixtures had narrow beam angles, but more recent fixtures have a broader distribution of photons. High wattage HPS fixtures need to be mounted higher above the canopy than LED fixtures to achieve uniform distribution of photons.
7. Fixture size for shading in a greenhouse application.

Because LEDs can be cycled on and off over short intervals, there has been interest in rapid cycling of LED fixtures to improve plant growth. Unfortunately, high frequency flickering (10–10000 Hz) has been well-studied, and there is neither empirical nor theoretical evidence that this can be used to increase the quantum yield of photosynthesis. Plants appear to integrate light intensity for photosynthesis^61,62^. However, some recent evidence indicates that longer-term cycling of LEDs (minute to hours) can alter plant shape (hypocotyl length) and color (anthocyanin synthesis)^63^.

## Summary

- Blue LEDs are now 93% efficient, phosphor-converted “whites” are 76% efficient, and reds are 81% efficient when run at the near-optimal conditions of 100 mA mm^−2^ and a junction temperature of 25 °C.
- Both junction temperature and drive current density will affect the photon efficacy of LEDs, and in general, the most efficient LED fixture will run their LEDs at low drive currents. However, a lower drive current results in a lower photon output per LED, and the resulting fixture will require many LEDs to achieve a high photon output and thus will be more expensive.
- Broad spectrum distribution of photons is useful for diagnosis of plant disorders. Broad spectrum lighting is not necessarily beneficial for photosynthesis or plant growth. Unique spectra, selectively applied during specific stages of the life cycle, can, however, have a beneficial effect on plant shape and development.
- The calculations in this paper show current possible performance levels of LED fixtures of 3.4 µmol J^−1^ for white + red fixtures, and 4.1 µmol J^−1^ for blue + red fixtures. These values are significantly higher than current typical values of 2–3 µmol J^−1^.

Although fixture efficacy is paramount, timing and angular delivery of photons to photosynthetic tissues, spectrum, and intensity also determine the effectiveness of the photon delivery system. Efficient lighting is then coupled with optimal temperature, humidity, nutrition, plant water potential, atmospheric carbon dioxide concentration, delivery of oxygen to root surfaces, and genetics. Both NASA and the USDA are funding research at universities to optimize these factors and improve the economic potential of electric lighting in in controlled environments: https://cubes.space/; https://www.hortlamp.org/index.html.
